# Case Report: Temozolomide Treatment of Refractory Prolactinoma Resistant to Dopamine Agonists

**DOI:** 10.3389/fendo.2021.616339

**Published:** 2021-03-12

**Authors:** Hao Tang, Yijun Cheng, Jinyan Huang, Jianfeng Li, Benyan Zhang, Zhe Bao Wu

**Affiliations:** ^1^ Department of Neurosurgery, Center of Pituitary Tumor, Ruijin Hospital, School of Medicine, Shanghai Jiao Tong University, Shanghai, China; ^2^ State Key Laboratory of Medical Genomics, Ruijin Hospital, School of Medicine, Shanghai Jiao Tong University, Shanghai, China; ^3^ Department of Pathology, Ruijin Hospital, School of Medicine, Shanghai Jiao Tong University, Shanghai, China

**Keywords:** resistance, dopamine agonist, PABPC1, temozolomide, prolactinoma

## Abstract

Therapeutic agents for refractory prolactinomas that are resistant to dopamine agonists (DAs) are troublesome, and surgery often only removes a large part of the tumor without complete remission. Among the various second-line treatment regimens, the treatment effect of the alkylating agent temozolomide (TMZ) is only effective for approximately half of patients; however, complete remission is rare. Here we report a patient with prolactinoma who was resistant to high-dose cabergoline (CAB) treatment, demonstrating a continuous increase in both the tumor volume and the prolactin (PRL) level. Given that this case is a refractory prolactinoma, the patient underwent two transsphenoidal approach (TSA) surgeries. The pathological analysis indicated that the Ki-67 index increased significantly from 3% to 30%, and the expression levels of DRD2 and MGMT were low. Finally, TMZ treatment was recommended. A total of six cycles of TMZ standard chemotherapy shrank the tumor volume and the tumor disappeared completely. During the 6-month follow-up period, the tumor did not relapse again, and the PRL level was also normal. RNA sequencing and DNA whole genome sequencing were performed on this prolactinoma specimen, revealing 16 possible gene mutations, including a missense mutation of the PABPC1 gene. Additionally, the copy number variation analysis results showed that several chromosomes had copy number gains compared to the matched peripheral blood sample. In this case, low expression of DRD2 and high proliferation led to resistance to CAB, whereas low MGMT expression contributed to sensitivity to TMZ treatment. The results of genome sequencing still need further investigation at the molecular level to explain the tumor aggressiveness and high sensitivity to TMZ.

## Introduction

Pituitary adenomas are the third most common intracranial tumor. The majority are considered to be benign, but approximately 35% are invasive (1, 2). Prolactinoma accounts for approximately 40% of pituitary adenomas, the majority of which can be treated with dopamine agonists (DAs), such as cabergoline (CAB) and bromocriptine (BRC). However, approximately 10-20% are resistant and refractory cases (3, 4), lacking effective treatments. A small number of tumors continue to enlarge significantly under high-dose DA treatment. However, these tumors are rarely reported, and the underlying mechanism is still unknown. To date, major studies have surmised that the resistance of prolactinoma to DAs may be due to the decrease in dopamine 2 receptor (DRD2) expression in tumor cells (5).

Temozolomide (TMZ) is a DNA alkylating agent that can cause base mismatches, which can trigger a futile DNA repair cycle. Eventually, the DNA strand breaks, which leads to cell death (6). TMZ has an effective stabilization rate of 27% for invasive and refractory pituitary tumors (7). TMZ is especially effective for 50% of patients with refractory prolactinomas who have failed conventional treatments, and increases the overall survival rate (7). However, there are fewer patients who achieve complete remission, and drug resistance and recurrence rates remain high (8, 9). In a systematic review of 42 patients with prolactinoma (or carcinoma) using TMZ treatment, only one patient with prolactin-secreting carcinoma and two patients with prolactinoma exhibited complete remission (10).

This study presents a case report of prolactinoma that is resistant to DAs and exhibits aggressive growth. Treatment with TMZ has achieved good and even significant antitumor effects. In addition, genomics and other research methods were used to investigate the underlying mechanism of drug therapy.

## Case Description

### History

**Figure 1:**
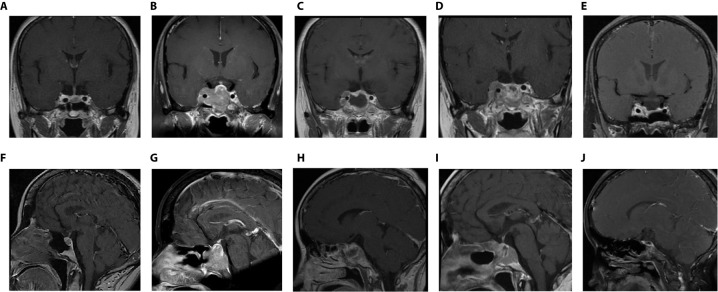
Coronal- and sagittal- enhanced MR images in various periods **(A, B)**: Before the first operation (5 years after DA treatment); **(C, D)**: 2 years after the first operation (before the second operation); **(E, F)**: The day after the second operation; **(G, H)**: 1 month after the second operation (the eve of TMZ treatment); **(I, J)**: 6 months after TMZ treatment.

A 40-year-old woman attended the endocrinology department due to menstrual disorders and lactation. Hormonal examination revealed a prolactin (PRL) of 105 ng/ml. A microadenoma in the sellar region was found on MR images with a size of approximately 0.5 * 0.5 * 0.5 cm. She was advised to take BRC 7.5 mg/d for the first 18 months, and the symptoms disappeared. Later, due to the incomplete control of PRL, she switched to CAB, gradually increasing and maintaining the dose at 2 mg/w. Two years after regular medication, the PRL level was reduced to 39 ng/ml (**Figure S1**), and the tumor appeared to shrink (0.4 * 0.4 * 0.3 cm in size) on MR images. However, over the next three years, she took the drugs, and the patient found that the PRL level gradually increased (133 ng/ml). However, the microadenoma exhibited an increasing trend (0.9 * 0.8 * 0.7 cm, **Figures 1A, B**), although the dose of CAB increased to 4 mg/w (**Figure S1**). Unfortunately, this patient often experienced dizziness and gastrointestinal discomfort with increased drug doses, so she was transferred to our Center of Pituitary Tumor for surgery. The center diagnosed this case of prolactin microadenoma resistant to DAs.

After careful consideration, at the age of 45 years, the patient underwent neuroendoscopic transsphenoidal pituitary adenoma resection. One week after the operation, the PRL level dropped to 54 ng/ml. The pathology confirmed pure PRL adenoma. Six months after the operation, the patient was re-examined by MR scan, and no obvious residual tumor was found. However, the PRL level increased to 89 ng/ml (**Figure S1**). After this follow-up visit, it was recommended that the patient continued to take CAB at the original tolerable dose (2 mg/w).

Unfortunately, the drugs were no longer able to inhibit tumor growth. Two years after regular medication, the dose of CAB was gradually increased to 4 mg/w. The patient was re-examined by MR, which revealed that the tumor continued to increase to approximately 3 * 3.2 * 2.6 cm in size with right cavernous sinus invasion (**Figures 1C, D**). The PRL level continued to increase with a peak level of 4902 ng/ml (**Figure S1**). In addition, she complained of visual field defects. After discussion with a multidisciplinary team (MDT), a diagnosis of refractory and aggressive prolactinoma was made. The second transsphenoidal pituitary tumor resection was performed to decompress the optic nerve.

New MR was conducted one day and one month after the second operation, showing remnant and rapidly relapsed tumors in the right cavernous sinus and sellar region, respectively (**Figures 1E–H**). The tumor size increased from 1 * 1.2 * 2 cm to 1.8 * 2 * 2.3 cm. The serum PRL level remained high (2066 ng/ml) (**Figure S1**). Based on the immunohistochemical (IHC) staining results of both tumor specimens collected from two operations subsequently (DRD2, MGMT and the change of Ki67, detailed in the “Pathology” section below), with consent, the patient was recommended to stop CAB and switch to standard chemotherapy with TMZ (240 mg × 5 d for the first month, 320 mg × 5 d per month for 2-6 months) for a total of 6 months. After 3 months of taking TMZ medicine, the tumor was significantly suppressed (0.8 * 0.7 * 0.4 cm). By the 6-month point of TMZ treatment, the tumor had completely disappeared (**Figures 1I, J**) and the PRL level returned to the normal level (18 ng/ml) (**Figure S1**).

The patient was followed up for another 6 months. The tumor did not reappear and the PRL level was also in the normal range (19 ng/ml).

### Pathology

**Figure 2:**
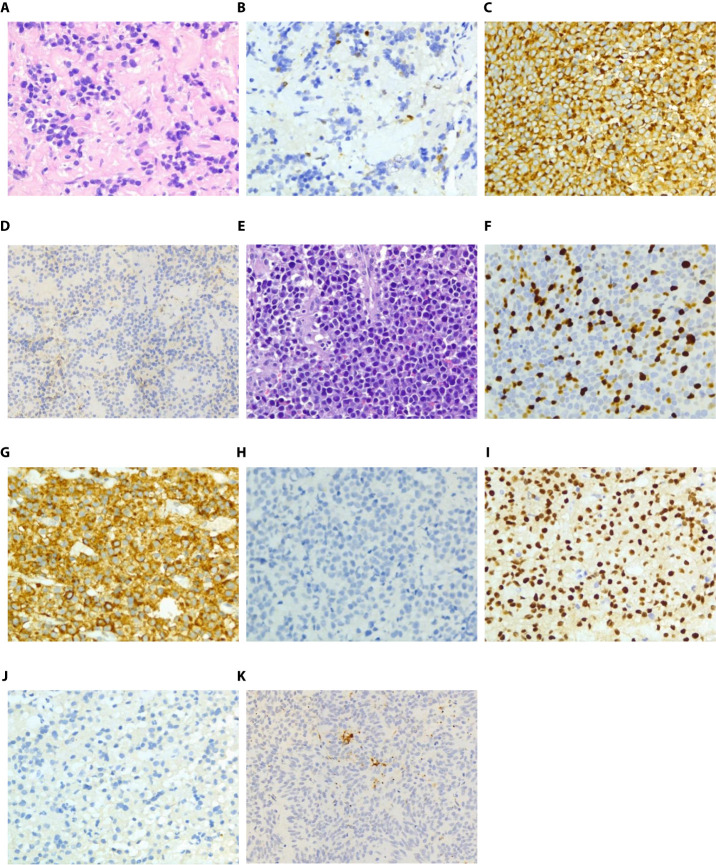
Pathological images of specimens after the first and second operations **(A)**: HE staining of specimens after the first operation; **(B)**: Ki-67 immunohistochemical staining of specimens after the first operation; **(C)**: PRL immunohistochemical staining of specimens after the first operation; **(D)**: DRD2 immunohistochemical staining of specimens after the first operation; **(E)**: HE staining of specimens after the second operation; **(F)**: Ki-67 immunohistochemical staining of specimens after the second operation; **(G)**: PRL immunohistochemical staining of specimens after the second operation; **(H)**: GH immunohistochemical staining of specimens after the second operation; (**I)**: Pit-1 immunohistochemical staining of specimens after the second operation; **(J)**: MGMT immunohistochemical staining of specimens after the second operation; **(K)**: DRD2 immunohistochemical staining of specimens after the second operation. Magnification: **(A–C, E–J)**: ×400; D&K: ×200.

Pathology of the first operation specimen: pituitary adenoma (**Figures 2A–D**, partial display). HE staining (**Figure 2A**); immunohistochemical (IHC) staining: Ki67 (3%+) (**Figure 2B**), PRL (+) (**Figure 2C**), ACTH (-), TSH (-), LH (-), FSH (-), GH (-), DRD2 (-) (**Figure 2D**), reticular fiber (-).

Pathology of the second operation specimen (**Figure 2E-K**, partial display): HE staining (**Figure 2 E**); IHC: Ki67 (30%+) (**Figure 2F**), PRL (+) (**Figure 2G**), ACTH (-), GH (-) (**Figure 2H**), FSH (-), LH (-), TSH (-), pit-1 (+) (**Figure 2 I**), MGMT (-) (**Figure 2J**), DRD2 (-) (**Figure 2K**), reticular fiber (-).

The pathological results have several characteristics. One was pit-1 positive (**Figure 2I**) and PRL positive (**Figure 2G**) but GH negative (**Figure 2H**), indicating that it is a single prolactin cell source, not a mixed adenoma. Another is to compare the active mitosis and high proliferation index of the two specimens. Ki67 increased from 3% (**Figure 2B**) in the first operation to 30% in the second operation (**Figure 2F**). Finally, the expression of DRD2 (**Figures 2D, K**) and MGMT was very low or even nonexistent (**Figure 2J**).

### Genome Sequencing

In view of the patient’s response to drugs, that is, resistance to DAs and the high sensitivity to TMZ, the pharmacotherapeutic mechanism had to be illustrated. RNA sequencing and DNA whole genome sequencing (WGS) on specimens from the second operation were performed. Genomic DNA and total RNA obtained from the patient were extracted using an AllPrep DNA/RNA Mini Kit (Qiagen) and subjected to paired-end (2 × 150 bp) sequencing on a NovaSeq platform (Illumina) according to the manufacturer’s protocol. WGS with an average read depth of 60× was analyzed using GATK (11) and VarScan2 (12). RNA-seq data were analyzed using STAR (13), salmon (14) and DESeq2 (15). RNA sequencing found that the number of DRD2 mRNA transcripts was very low (only 5 cycles, data not shown) compared to the other 23 prolactinoma specimens (15 resistant and 8 sensitive to DAs). WGS confirmed somatic mutations in FAM160B1, MYBPC1, CSPG4, GAN, CIRBP, PRX, SF3B1, PABPC1, CABS1, TENM3, DMXL1, HOXA3, CSGALNACT, PLEC, RBL2, and KIR2DL3 (**Table 1**). Among them, the missense mutation in PABPC1 (poly (A) binding protein cytoplasmic 1) is located at base 1424, exon 10. The mutation from guanine (G) to adenine (A) results in the conversion of the 475th amino acid of the expression protein from arginine (R) to glutamine (Q).

**Table 1:** 16 possible gene mutations identified with whole genome sequencing.

Gene	Chromosome	Location	Ref → Seq	Tumor variant frequency	Coding	Amino acid change
FAM160B1	chr10	114846067	A → G	0.2903	c.1183A>G	p.M395V
MYBPC1	chr12	101652686	A → C	0.2075	c.1460A>C	p.K487T
CSPG4	chr15	75689920	A → C	0.25	c.1145T>G	p.L382R
GAN	chr16	81362563	G → T	0.45	c.1038G>T	p.K346N
CIRBP	chr19	1272068	G → C	0.4444	c.519G>C	p.E173D
PRX	chr19	40394057	C → A	0.4348	c.4295G>T	p.G1432V
SF3B1	chr2	197402759	C → T	0.5	c.1874G>A	p.R625H
CABS1	chr4	70335766	G → A	0.2927	c.727G>A	p.D243N
TENM3	chr4	182751942	G → A	0.2895	c.3772G>A	p.A1258T
DMXL1	chr5	119129367	C → T	0.2031	c.1259C>T	p.P420L
PABPC1	chr8	100706893	G → A	0.3227	c.1424G>A	p.R475Q
HOXA3	chr7	27110570	A → C	0.2034	c.71T>G	p.F24C
CSGALNACT1	chr8	19406045	C → A	0.3871	c.1334G>T	p.G445V
PLEC	chr8	143917661	C → T	0.3438	c.12571G>A	p.D4191N
RBL2	chr16	53490280	A → C	0.25	c.3400A>C	p.N1134H
RBL2	chr16	53490281	A → C	0.2703	c.3401A>C	p.N1134T
KIR2DL3	chr19	54752514	C → T	0.6087	c.1021C>T	p.P341S

**Figure 3:**
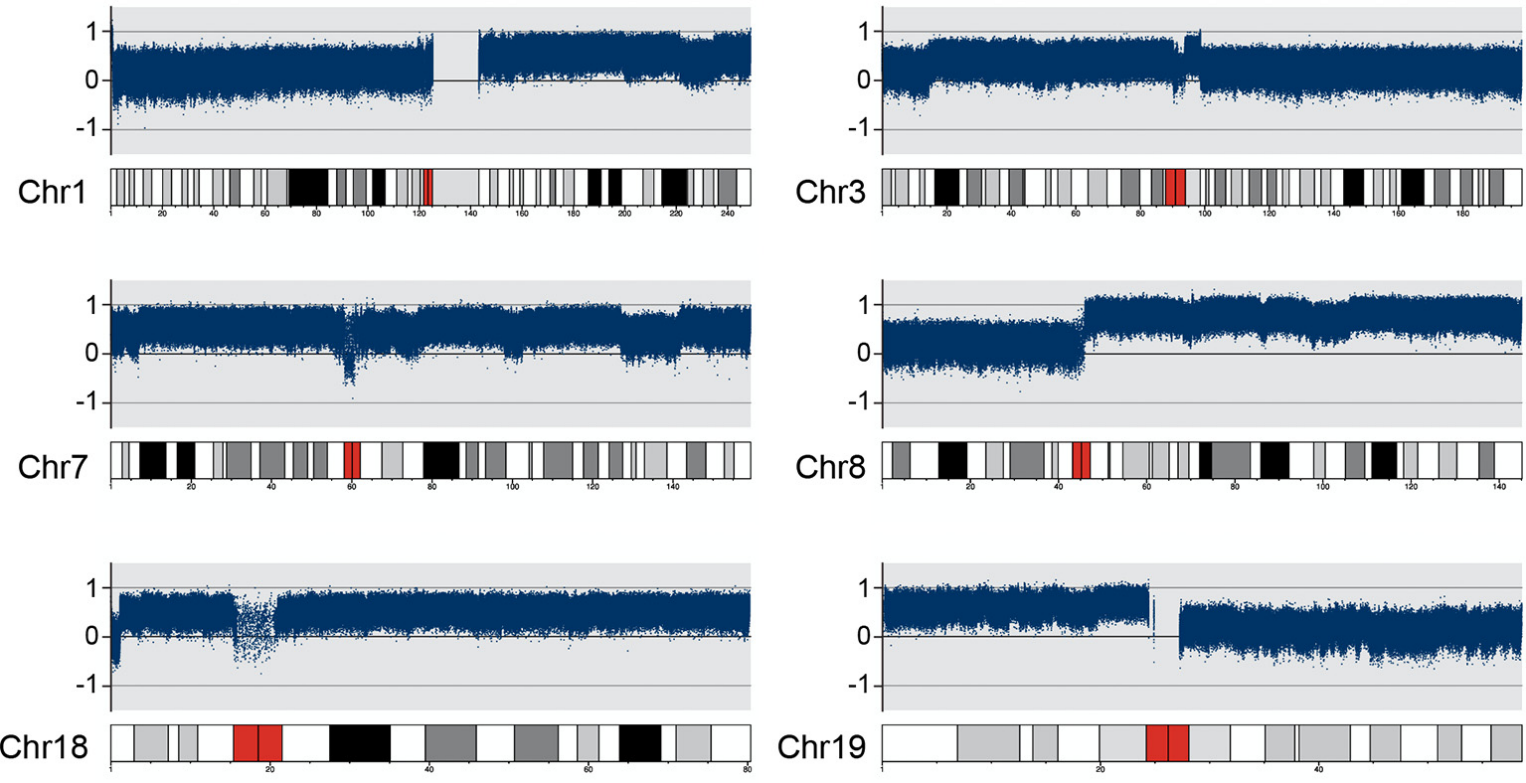
DNA copy number variation analysis results on chr1, chr3, chr7, chr8, chr18 and chr19 Several chromosomes had copy number gains compared to the matched PB (peripheral blood) sample. Chr1&8 have copy number gains on the long arm (q); Chr3&19 have copy number gains on the short arm (p); Chr7&18 have copy number gain on almost the entire chromosome.

On the other hand, copy number variation analysis results showed that several chromosomes had copy number gains compared to the matched peripheral blood sample, e.g., chr1, chr3, chr7, chr8, chr18 and chr19. Most of them (chr1, chr3, chr8 and chr19) had copy number gains on the long arm (q) or short arm (p), whereas there was almost one whole chromosome copy number gain on chr7 and chr18 (**Figure 3**).

## Discussion

It is well known that the effectiveness of DAs on prolactinoma is determined by the expression of DRD2 and that a decrease in DRD2 expression may play a critical role in DA-resistant prolactinoma (16–18). Our previous study (19) found that DRD2 expression was significantly different between resistant and sensitive prolactinomas, and even between aggressive and nonaggressive prolactinomas. In this study, we used immunohistochemical (IHC) staining, which is commonly used in pathology, to identify the expression level of specific protein molecules in cells. IHC staining of the patient’s tumor specimens revealed very low expression of DRD2, which probably explained the tumor’s resistance to high-dose CAB treatment. In addition, this tumor resistance might be related to the high degree of tumor aggressiveness. Ki67 expression obviously increased, reaching 30%, which combined with invasion of the right cavernous sinus, indicating that the tumor exhibited significant aggressiveness. The tumor volume even increased under high-dose CAB treatment. All the above results indicated that this was a refractory prolactinoma resistant to DA treatment.

TMZ has some effect on refractory prolactinomas or carcinomas, but the proportion of patients with complete remission is very low (10, 20). In the TMZ treatment cohort study of 166 aggressive pituitary tumors initiated by the European Society of Endocrinology (ESE) (20), 40 cases were aggressive, refractory prolactinoma or carcinoma, and 2 of these cases (5%) achieved complete remission after treatment. The article did not disclose the detailed PRL or imaging changes of the cases. In another review (10), among the 42 cases included, there were 23 patients with prolactinoma and 19 patients with prolactin-secreting pituitary carcinoma, of whom 3 patients (3/42, 7%) had their PRL levels reduced to normal after TMZ treatment. However, the original reports of the two prolactinoma cases with complete remission did not show the detailed size changes and imaging data of the tumors before and after treatment (21, 22). In addition, a patient with prolactin-secreting pituitary carcinoma who achieved complete remission was reported in detail (23). He was an elderly male (> 70 y) and had metastatic lesions in the cerebellopontine angle and cervical spine in addition to a primary tumor in the sellar region. Three tumor lesions disappeared completely after 18 months of TMZ treatment. Our present case cannot be defined as prolactin-secreting pituitary carcinoma because there was no intracranial metastasis. This tumor exhibited a complete response to TMZ treatment with a biological cure.

Obviously, the effect of TMZ treatment has a strong correlation with the expression of MGMT in glioma (24, 25). Specifically, the lower the degree of MGMT expression, the better the tumor’s response to TMZ treatment. However, in pituitary tumors, there is controversy regarding the relationship between MGMT expression and tumor response to TMZ treatment. MGMT protein expression was very low in this case, indicating that refractory tumor cells were highly sensitive to TMZ treatment.

DNA whole genome sequencing showed 16 possible gene mutations, including the missense mutation of the PABPC1 gene. Wang Q *et al.* (26) reported that PABPC1 is downregulated in highly malignant gliomas and that high expression of PABPC1 is significantly related to a better prognosis and is related to the biological process of translation. It is reasonable to believe that the aggressiveness and proliferation index of pituitary tumors may also be negatively correlated with the biological activity of PABPC1 protein. Of course, the specific mechanism still needs further exploration in *in vitro* and *in vivo* experiments.

## Conclusion

The above reports on a case of refractory prolactinoma that was significantly aggressive and resistant to DA treatment, but was highly sensitive to the alkylating agent TMZ. Low DRD2 expression and high proliferation lead to resistance to BRC and CAB, while low expression of MGMT contributes to sensitivity to TMZ treatment. The results of genome sequencing still requires further investigations to explain the underlying molecular mechanisms of tumor aggressiveness and high sensitivity to the alkylating agent TMZ.

## Data Availability Statement

The original contributions presented in the study are included in the article/**Supplementary Material**. Further inquiries can be directed to the corresponding author.

## Ethics Statement

Ethical review and approval were not required for the study on human participants in accordance with the local legislation and institutional requirements. The patients/participants provided their written informed consent to participate in this study. Written informed consent was obtained from the individual(s) for the publication of any potentially identifiable images or data included in this article.

## Author Contributions

HT wrote the first draft of the manuscript. YC and ZBW contributed to the design and writing. JH and JL provided the genome sequencing analysis. BZ provided the pathological figures. HT provided the other figures. All authors contributed to the article and approved the submitted version.

## Funding

This work was supported by the Shanghai Municipal Science and Technology Commission 18XD1403400 (ZBW), Program of Shanghai Academic Research Leader (ZBW), Shanghai Training 641 and Support Program for Outstanding Young Medical Talents 642 (ZBW), and Ruijin Youth NSFC Cultivation Fund (2019QNPY01048).

## Conflict of Interest

The authors declare that the research was conducted in the absence of any commercial or financial relationships that could be construed as a potential conflict of interest.
