# Supplementary figures and images for: Case Report: Temozolomide Treatment of Refractory Prolactinoma Resistant to Dopamine Agonists
Source: Front Endocrinol (Lausanne). 2021 Mar 12;12:616339. doi: 10.3389/fendo.2021.616339 (PMC7996095; doi:10.3389/fendo.2021.616339)

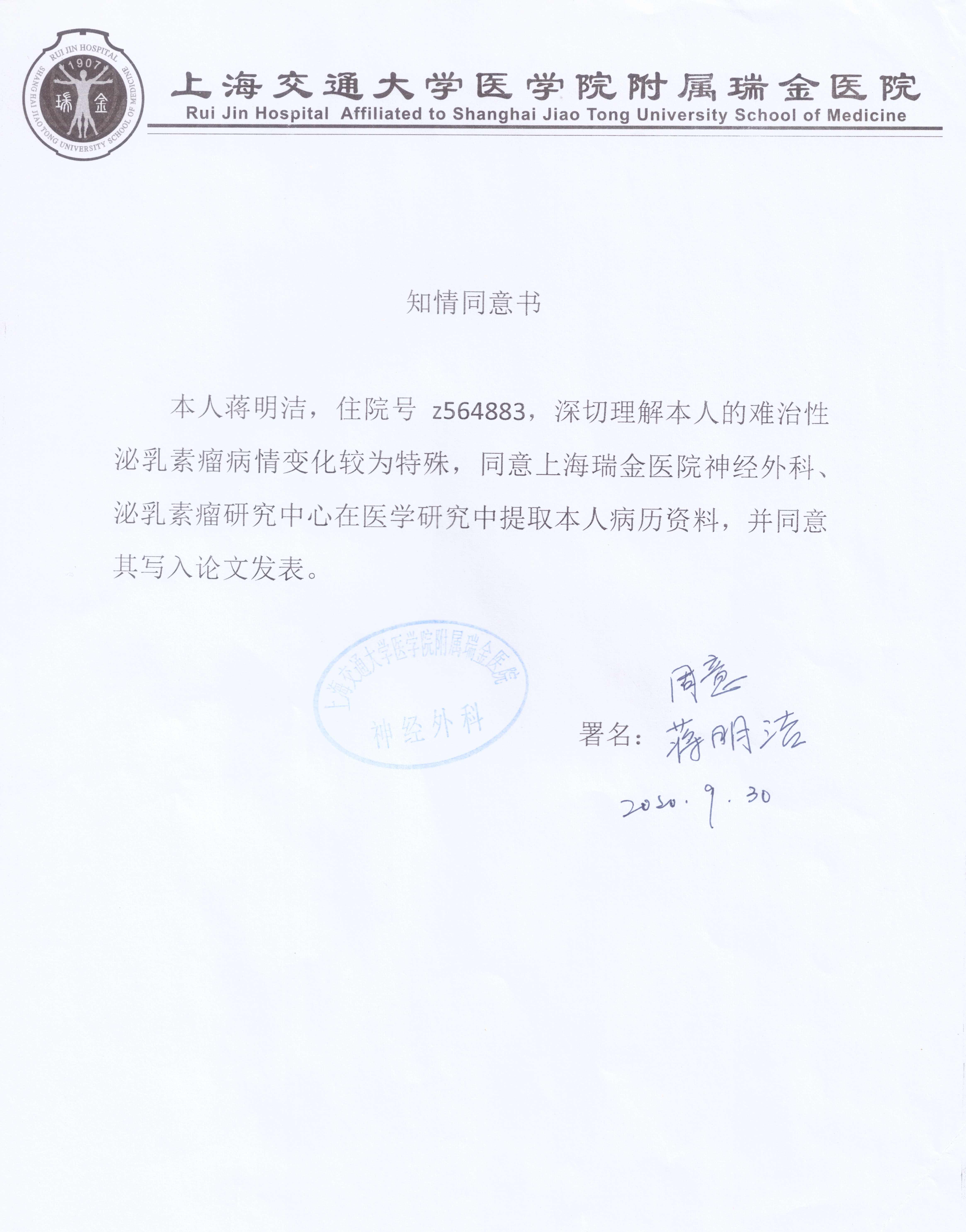

Supplement: Supplementary file 2 [file Image_1.jpeg]

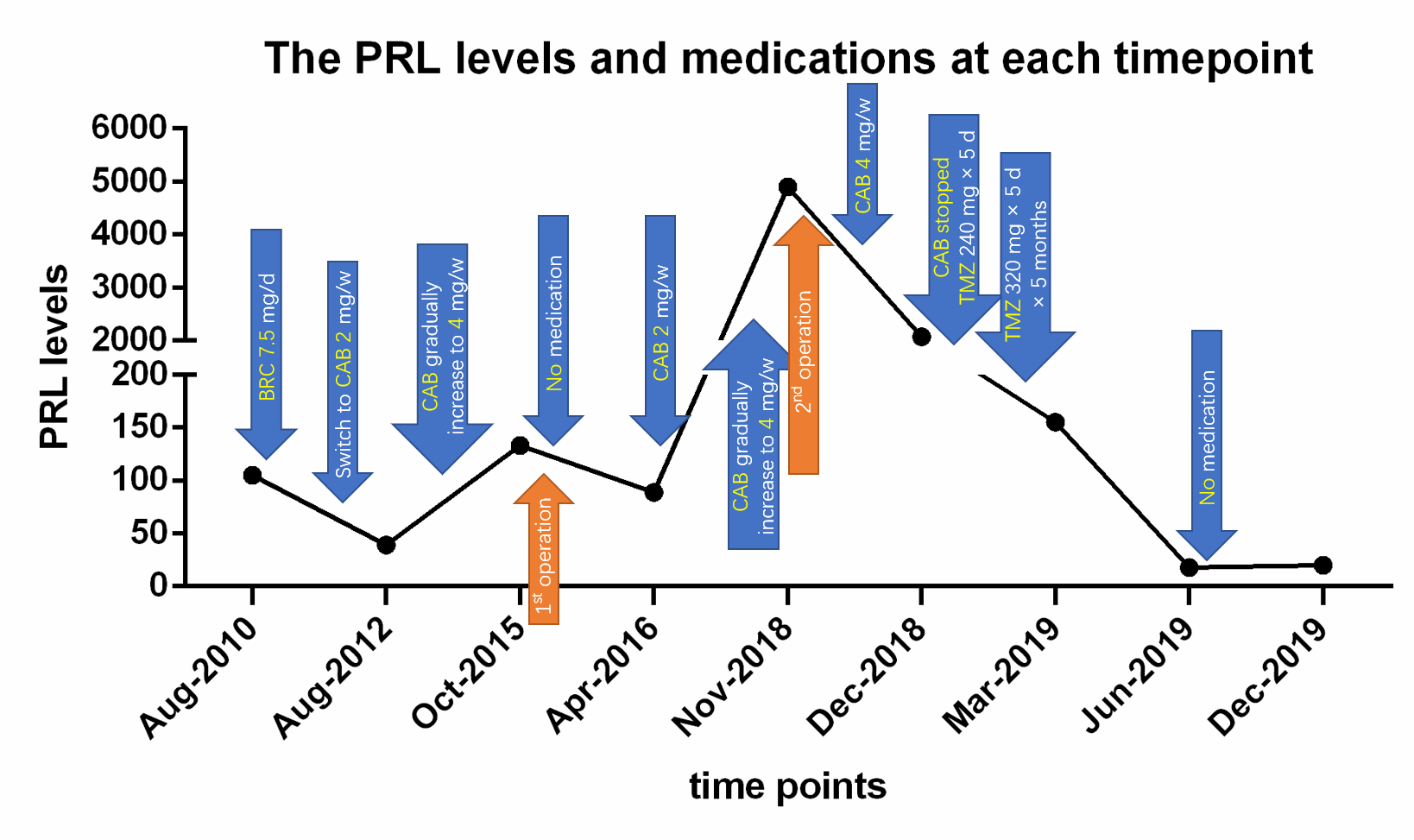

Supplement: Supplementary file 3 [file Image_2.tif]
